# Preconcentration of Fe(III), Co(II), Ni(II), Cu(II), Zn(II) and Pb(II) with ethylenediamine-modified graphene oxide

**DOI:** 10.1007/s00604-015-1629-y

**Published:** 2015-09-07

**Authors:** Beata Zawisza, Anna Baranik, Ewa Malicka, Ewa Talik, Rafał Sitko

**Affiliations:** Institute of Chemistry, University of Silesia, ul. Szkolna 9, 40-006 Katowice, Poland; Institute of Physics, University of Silesia, ul. Uniwersytecka 4, 40-007 Katowice, Poland

**Keywords:** Modified graphene oxide, Scanning electron microscopy, Energy-dispersive spectrometry, Sorbent, Environmental analysis, Micro-solid phase extraction, X-ray fluorescence spectrometry

## Abstract

We describe a novel solid phase sorbent that was synthesized by coupling graphene oxide (GO) to ethylenediamine (EDA). This nanomaterial (referred to as GO-EDA) is capable of adsorbing the ions of iron, cobalt, nickel, copper, zinc and lead. The ethylenediamine-modified graphene oxide was characterized by X-ray photoelectron spectroscopy, scanning electron microscopy and Fourier transform infrared spectroscopy. The analytical procedure relies on (a) sorption of metal ions on GO-EDA dispersed in aqueous samples; (b) filtering, and (c) direct submission of the filter paper to energy-dispersive X-ray fluorescence spectrometry. This kind of dispersive micro-solid phase extraction was optimized with respect to pH values, concentration of GO-EDA, contact time, and the effects of interfering ions and humic acid on recovery of determined elements. Under optimized conditions, the recoveries of spiked samples range from 90 to 98 %. The detection limits are 0.07, 0.10, 0.07, 0.08, 0.06 and 0.10 ng mL^−1^ for Fe(III), Co(II), Ni(II), Cu(II), Zn(II) and Pb(II), respectively. The method has a relative standard deviation of <6 %, and its accuracy was verified by analysis of two standard reference materials [LGC6016 (estuarine water) and BCR-610 (groundwater)]. It was successfully applied to the determination of trace amounts of these metal ions in water samples.

**Electronic supplementary material:**

The online version of this article (doi:10.1007/s00604-015-1629-y) contains supplementary material, which is available to authorized users.

**Graphical Abstract:**
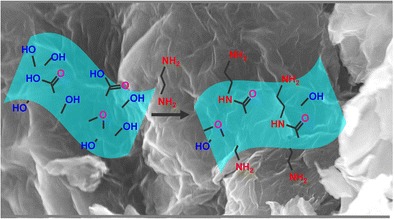
Graphene oxide was coupled to ethylenediamine in order to obtain an effective sorbent (GO-EDA) for preconcentration of Fe(III), Co(II), Ni(II), Cu(II), Zn(II) and Pb(II) from environmental water samples

## Introduction

The pollution of environment, particularly by heavy metals occurs due to various reasons included natural ones such as leaching of rocks, minerals and soils containing different concentration of these elements, and industrial reason like mining, discharge of industrial pollutants. Although some of elements are needed for human body and environment. However, some of heavy elements can be very toxic or even carcinogenic when consumed or exposured in larger amounts over a long time. Hence, it is of great importance to the determination of heavy metals in different type of environmental samples particularly in water from industrial area.

Popular techniques in the analytical chemistry using for the preconcentration of heavy metal ions include the precipitation, the membrane filtration, the extraction, the sorption, and the ion exchange. Especially, sorption techniques have been widely used because they are simple, economical, and cost-effective. Some sorbents, such as activated carbon [1], C18 [2], polymers [3], silica gel [4], various resins [5], polyurethane foams [6] and biomass [7] suffer from low sorption capacities, efficiencies or their applicability, which is often restricted only to limited number of analytes. Nanomaterials play an important role to solve this problem because of their high surface area, enhanced active sites, and additionally the presence of numerous functional groups on their surfaces [8–10]. In the literature the synthesis, application of carbon nanotubes, graphene, graphene oxides [11, 12] and interaction mechanism between metal ions and graphene oxide [13–15] as well as the wide use of carbon nanomaterials in solid-phase extraction (SPE) and solid-phase microextraction (SPME) is described [16]. The applications of carbon nanotubes (CNTs) and graphene (G) in sample preparation have greatly increase in recent years. CNTs including single-walled nanotubes (SWCNTs) and multi-walled nanotubes (MWCNTs) have been widely studied regarding their potential environmental application as superior adsorbents for heavy metals [17, 18] and organic compounds [19, 20] during solid-phase extraction and wastewater treatment. However, the selectivity of the raw or oxidized-MCWNTs for SPE is quite limited, especially for metal ions. MWCNTs modified with some organic compounds are expected to be more selective than untreated and oxidized-MWCNTs for the solid-phase extraction of metal ions. Graphene, another carbon nanomaterial, has a very large specific surface area [21] suggesting a high adsorption capacity. It arises from the unique nanosheet morphology of graphene. In graphene unlike CNTs both surfaces of the planar sheet are accessible for the molecule adsorption. The nanosheet structure of graphene is also conducive to fast adsorption equilibrium. G can be easily modified with functional groups, especially via graphene oxide which has many reactive groups. The modification of the nanometer-sized oxides increases the number of binding sites able to interact with metal ions. The modification of these materials can also change the binding sites in order to enhance the uptake of metal ions [22, 23]. Li et al. studied the simultaneous removal of fulvic acid and Cu(II) from aqueous solution by graphene oxide nanosheets decorated with Fe_3_O_4_ nanoparticles (GO/Fe_3_O_4_) [24]. The results showed that GO/Fe_3_O_4_, which combined the high sorption capacity of graphene oxide and the separation facility of Fe_3_O_4_ can be used as the effective sorbent for the rapid removal of inorganic and organic pollutants from water samples. Modification of the graphene oxide may further enhance the capacity and selectivity of SPE methods. 3-(1-Methyl-1H-pyrrol-2-yl)-1H-pyrazole-5-carboxylic acid (MPPC) and graphene oxide were used in a glass columnas as chelating reagent and as adsorbent respectively prior to the determination of manganese and iron ions [25]. In the paper [26] graphene oxide was covalently bonded to silica by coupling the amino groups of spherical aminosilica and the carboxyl groups of graphene oxide. Graphene oxide covalently bonded to silica (GO@SiO_2_) was successfully applied in the preconcentration and the determination of copper and lead. Liu et al. developed graphene-bound silica (G@silica) and graphene oxide-bound silica (GO@silica) as SPE adsorbents. Because graphene and graphene oxide have a different polarity non-polar G@silica and polar GO@silica could be used for reversed-phase and normal-phase SPE, respectively [27]. Graphene oxide-coated SiO_2_ particles prepared through electrostatic assembly was used by others authors for the selective extraction of hemoglobin from human blood [28]. The few-layered graphene oxide nanosheets has been synthesized and used as adsorbents for the removal of Cd(II) and Co(II) from aqueous solutions [29]. The described method was a sensitive and accurate for the determination of manganese and iron at low concentrations in different water, food and biological samples. The method was characterized by low limit of detection, high efficiency and a high preconcentration factor (325) with relatively high stability of prepared sorbents against acids.

In this work, a solid phase sorbent: ethylenediamine-modified graphene oxide (GO-EDA) was synthesized to develop a dispersive micro-solid phase extraction (DMSPE) method for preconcentration/separation of Fe(III), Co(II), Ni(II), Cu(II), Zn(II) and Pb(II) prior to their determination by X-ray fluorescence spectrometry in natural water. A significant advantage of synthesized GO-EDA over CNTs is, among other things, fact that it can be readily obtained from graphite in most chemical laboratories. This fact, greatly promotes its wide application in preconcentration process at low cost. The developed DMSPE method is time efficient, simple, and reagent non-consuming in comparison with a classical SPE. Due to the fact, that the metal ions adsorbed onto GO-EDA are directly detected by XRF spectrometer the elution of analytes is unnecessary. In consequence, the use of solvents is eliminated, and the time of sample preparation as well as the source of contamination are reduced. The method has been applied for the determination of selected trace heavy metals in different type of water.

## Experimental

### Materials

Stock solutions (1 mg mL^−1^ of Fe(III), Co(II), Ni(II), Cu(II), Zn(II) and Pb(II)), nitric acid (65 %, Suprapur®), ammonium hydroxide solution (25 %, Suprapur®) were purchased from Merck (Darmstadt, Germany, www.**merck**group.com), graphite powder, N,N′- Dicyclohexylcarbodiimide (DCC, 99 %), ethylenediamine (EDA) and humic acid (HA) were purchased from Sigma-Aldrich (St. Louis, USA, www.**sigmaaldrich**.com), potassium permanganate (KMnO_4_), concentrated sulfuric acid (H2SO4), concentrated hydrochloric acid (HCl) and sodium nitrate (NaNO_3_) were purchased from POCh (Gliwice, Poland, www.**poch**.com).

The pH of the analyzed solutions was adjusted with 0.1 mol L^−1^ HNO_3_ and 0.1 mol L^−1^ NH_3_ · H_2_O. A multielement standard solution (10 μg mL^−1^ of Fe(III), Co(II), Ni(II), Cu(II), Zn(II) and Pb(II)) was prepared from the stock solutions (1 mg mL^−1^). All reagents were dissolved and diluted with high purity water obtained from Milli-Q system.

Certified reference material BCR-610 (ground water) was obtained from The Institute for Reference Materials and Measurements of Joint Research Centre (Geel, Belgium), LGC6016 (estuarine water) from LGC-Standards (Teddington, UK).

### Apparatus

The microstructural observations of the GO-EDA were conducted on the JEOL-7600F scanning electron microscope (SEM) equipped with the Oxford X-ray energy-dispersive spectrometer (EDS).

The chemical composition of GO-EDA was confirmed by the X-ray photoelectron spectroscopy (XPS). Photoelectron spectra were collected using the PHI 5700/660 Physical Electronic spectrometer with monochromated Al Kα radiation. The spectra were analyzed with a hemispherical mirror assuring an energy resolution of about 0.3 eV. Three hours after placing the samples in situ at 10–10 hPa vacuum, their surface was clean enough for measurements. The binding energy in the range −2 to 1400 eV and the core-level characteristic peaks for C1s have been measured. The background was subtracted using the Tougaard’s approximation.

The elemental composition was determined using the energy-dispersive X-ray fluorescence spectrometry (EDXRF). The spectra were collected using spectrometer Epsilon 3 (Panalytical, Almelo, The Netherlands, www.**panalytical**.com) with the Rh target X-ray tube with 50 μm Be window and max. power of 9 W. The spectrometer is equipped with thermoelectrically cooled silicon drift detector (SDD) with 8 μm Be window and resolution of 135 eV at 5.9 keV. The X-ray tube was operated at 5 kV and 1000 μA, and spectra were collected in helium atmosphere without primary beam filter.

### Synthesis of graphene oxide

Graphite oxide was prepared using modified Hummers’ method through the oxidation of natural graphite powder [30]. Briefly, 3 g of graphite was mixed with 1.5 g of NaNO_3_ and 69 mL of H_2_SO_4_. The mixture was cooled to 0 °C using an ice bath, and then 9 g of KMnO_4_ was added using small portions, to keep the extraction time of 5 min temperature below 20 °C. The obtained mixture was warmed, and constantly stirred for 12 h at 35 °C. After that, the reaction mixture was cooled to room temperature and poured into baker with 400 mL of ice and 3 mL of 30 % H_2_O_2_. In the next step, the solid was separated from the reaction mixture, using a centrifuge (5040 rcf). The collected solid was purified with 10 % HCl, 5 % HCl, and water. While being washed, the solid was suspended by ultra-sonication, and then it was centrifuged. The final product was dried at 100 °C.

### Synthesis of ethylenediamine-modified graphene oxide

0.5 g of graphene oxide was suspended in 20 mL of ethylenediamine under stirring and heating, then 0.7 g of N,N′-dicyclohexylcarbodiimide was added into the suspension and refluxed for 48 h at 60 °C. The final product (GO-EDA) was filtered off, washed with ethanol and dried in an oven at 80 ° C for 8 h.

### Preparation of the water suspension of ethylenediamine-modified graphene oxide 10 mg mL^−1^

250 mg of GO-EDA was put into 25 mL flasks, and filled with water up to the mark. In order to achieve homogenization, the suspension of GO-EDA was placed for 15 min in an ultrasonic bath directly before the use.

### Preconcentration procedure - dispersive micro-solid phase extraction

200 μL of 10 mg mL^−1^ suspension of GO-EDA was added to 50 mL of aqueous sample. The pH of the solution was adjusted to 8 with 0.1 mol L^−1^ HNO_3_, and/or 0.1 mol L^−1^ NH_3_. Next, the mixture was stirred by a magnetic stirrer for 5 min. After that, the sample was collected onto the Millipore filter (mixed cellulose esters, HA, 0.45 mm pore size) of 5 mm in diameter using a vacum filtration assembly. The loaded filter was dried under an IR heater. After that, the loaded filter was placed between two 6.0 μm-thick Mylar X-ray foils mounted in a special sample holder.

A blank sample was prepared using high purity water obtained from the Milli-Q system instead of the analyzed solution, according to the procedure described above.

### Preparation of real samples

The real water samples were filtered through a Millipore membrane filter of 0.45 mm pore size, and then stabilized by acidification with concentrated nitric acid to achieve a pH of 2. The water samples were stored at 4 °C in polyethylene bottles. In order to determine Fe(III), Co(II), Ni(II), Cu(II), Zn(II) and Pb(II), the analytes were preconcentrated using the dispersive micro-solid phase extraction procedure described above.

## Results and discussion

### The characterization of synthesized ethylenediamine-modified graphene oxide

**Fig. 1:**
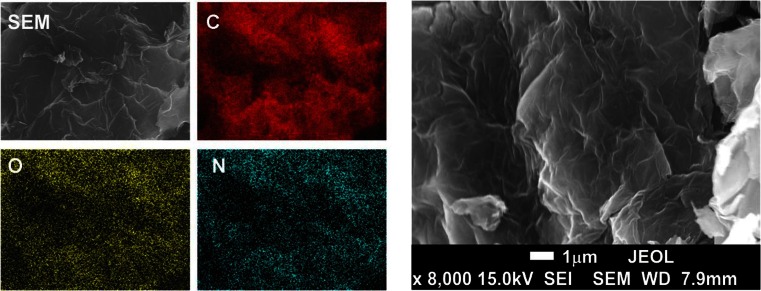
SEM/EDS images of synthesized ethylenediamine-modified graphene oxide and reveals the correlation between distribution of C, O and N elements on the ethylenediamine-modified graphene oxide surface

The synthesized ethylenediamine-modified graphene oxide was characterized by the scanning electron microscope analysis. Figure 1 shows the SEM/EDS images of synthesized ethylenediamine-modified graphene oxide and reveals the correlation between distribution of C, O and N elements on the GO-EDA surface. The highly wrinkled structure of the ethylenediamine-modified graphene oxide nanosheets can result in a large surface area and high extractive capacity.

**Fig. 2:**
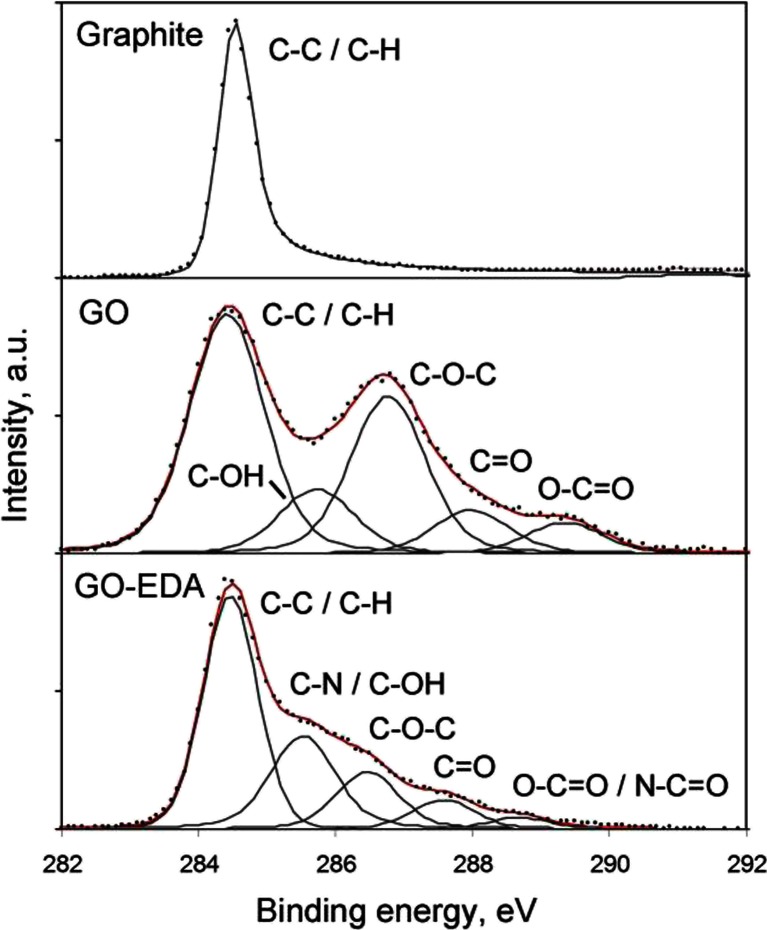
The high-resolution C1s spectra for graphite, synthesized graphene oxide and ethylenediamine-modified graphene oxide

The chemical composition of ethylenediamine-modified graphene oxide was also confirmed by X-ray photoelectron spectroscopy. The high-resolution C1s spectra presented in Fig. 2. indicate, that there are significant differences between the C1s peak for graphite, and synthesized GO and GO-EDA. The XPS spectrum of graphite shows the main peak at 284.5 eV due to the graphitic carbon, whereas spectrum of GO reveals also other four peaks at 285.8, 286.8, 288.1 and 289.4 eV assigned to C-OH, C-O-C, C=O and O-C=O. The C1s spectrum of GO-EDA was deconvoluted into five peaks at 284.5 eV (C-C and C-H), 285.6 eV (C-OH, C-N), 286.5 eV (C-O-C), 287.6 eV (C=O), and 288.7 eV (O-C=O and N-C=O) [31–33]. Because of the smaller electronegativity difference between C and N compared to between C and O, the peaks for C-N/C-OH and O-C=O/N-C=O are observed at a lower binding energy (in comparison with C-OH and O-C=O in GO) [34]. Summarizing, the significant difference C1s peaks (in the shape, intensity and the maximum position) indicates the successful modification of graphene oxide nanosheets with ethylenediamine.

**Fig. 3:**
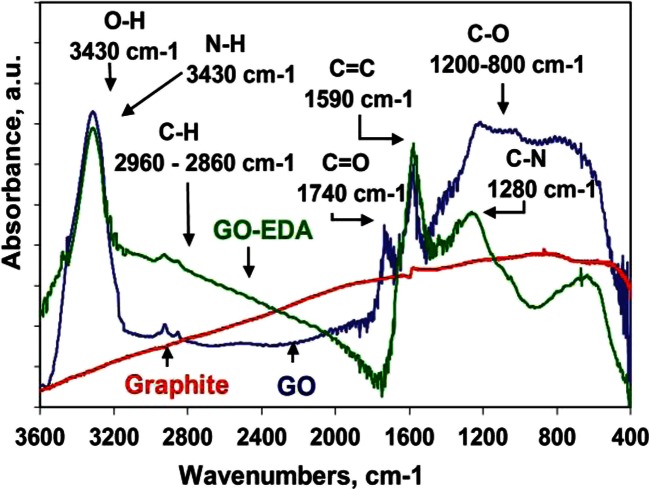
Fourier transform infrared (FTIR) spectra of graphite, graphene oxide and ethylenediamine-modified graphene oxide

The ethylenediamine-modified graphene oxide was identified by Fourier transform infrared spectroscopy (FTIR). The FTIR spectrum of graphene oxide exhibited a strong absorption band at 1200–1250, because of the C-O stretching, and at 1590 cm^−1^ corresponding to the C=C stretching. After the extraction time of 5 min, a new band emerged at 1280 cm^−1^, corresponding to the C−N characteristic stretching vibrations. In addition, the presence of strong N−H stretching and bending vibrations at 3430 cm^−1^, and associated with C-H stretching bands (2860–2960 cm^−1^) demonstrated the successful modification of graphene oxide with ethylenediamine. [35]. FTIR spectra of graphite, graphene oxide and ethylenediamine-modified graphene oxide are shown in Fig. 3.

### Optimization of method

The following parameters were optimized: (a) sample pH value; (b) concentration of ethylenediamine-modified graphene oxide and (c) reaction/extraction time.

Respective data and Figures (S1-S3) are given in the Electronic Supporting Material. The following experimental conditions were found to give best results: (a) a sample pH value of 8; (b) a concentration of ethylenediamine-modified graphene oxide of 0.04 g L^−1^ and (c) a reaction/extraction time of 5 min.

### The effect of matrix elements

The effects of common coexisting ions in different types of water, such as Na(I), K(I), Mg(II), Ca(II), on the preconcentration of Fe(III), Co(II), Ni(II), Cu(II), Zn(II) and Pb(II) on ethylenediamine-modified graphene oxide were investigated. The recovery of the determined elements was investigated from the solutions containing Na(I), K(I), Mg(II) and Ca(II) at the concentration of 4–40 μg mL^−1^. The recoveries of determined elements in various amounts of matrix ions are given in the Figure S4 in Electronic Supporting Material. The major cations, which can be find in natural water have no negative influence on the determination of Fe(III), Co(II), Ni(II), Cu(II), Zn(II) and Pb(II), under the optimized conditions. The recoveries of determined elements were never worse than 90 %. High recoveries of determined elements result from the fact, that the sorption of alkaline metals is not occurred, and Na(I) and K(I) remain in the solution. Therefore, the developed method can be used in the presence of high concentration of alkaline metals. But it can be noted that magnesium and calcium can precipitate, inter alia as hydroxides in the solution of pH = 8.

In some types of water the concentration of Fe(III) can be high and in consequence it can influence on the determination of trace amounts of remaining elements. Thus, the influence of the high concentration of Fe(III) on the recovery of Co(II), Ni(II), Cu(II), Zn(II) and Pb(II) on GO-EDA was investigated in detail. The recovery of Co(II), Ni(II), Cu(II), Zn(II) and Pb(II) was studied from the solutions containing Fe(III) at the concentration of 0.02-2 μg mL^−1^. The obtained results (see the Electronic Supporting Material, Fig. S5) indicate that Fe(III), within studied range of the concentration, has no significant effect on the sorption and the determination of Co(II), Ni(II), Cu(II), Zn(II) and Pb(II) on ethylenediamine-modified graphene oxide in water samples.

Humic acid (HA), which is ubiquitous in natural environment, can effectively influence the transportation and removal of heavy metals. In recent studies, Hyung et al. [36, 37] reported that natural organic matter (NOM) such as inter alia HA interacted strongly with CNTs and formed stable complexes in aqueous solutions. These carbon nanomaterials readily interact with NOM upon contact [38]. As the result of these interactions, the surface properties of carbon nanomaterials, as well as the adsorption of environmental contaminants by CNTs would be altered. The strong complex abilities between HA and metal ions can also influence the interaction among heavy metals, humic acid and carbon nanomaterials. HA is expected to have influence on metal ion sorption by ethylenediamine-modified graphene oxide. The hydrophilic fractions of HA have various functional groups, such as hydroxyl, carboxyl, amine, phenol and quinine groups that can bind heavy metals. It should be also noted that the dissolved HA and GO-EDA-bound HA may influence the increase the sorption of metal ions through chemical complexation and electrostatic attraction, respectively. Generally, in acidic and neutral solutions, dissolved HA could enhance the sorption of heavy metals by nanomaterials due to the electrostatic attraction. The strong complexation ability of surface adsorbed HA with metal ions can result in the increase of metal ion sorption on carbon nanomaterials. Nevertheless, in alkaline solutions, the most of HA can exist freely in solution, and then it can form soluble complexes of HA-metal ions, and consequently it can lead to the decrease of metal ion sorption on nanomaterials. Broadly speaking, heavy metals co-exist in the environment and HA, where they may affect each other’s behavior. Therefore, it is of great practical importance to study the effects of HA on the preconcentration of heavy metals by GO-EDA in water samples.

The influence of HA was investigated in the concentration range from 0.1 to 1 μg mL^−1^. The results of this investigation are presented in the Fig. S6 in Electronic Supporting Material. No obvious difference was found in the presence or absence of HA. The data obtained indicate that spiked water with humic acid gives good recoveries for each determined metal (ca. 92 %). Only slight overestimation of the recoveries for cobalt, zinc and cobalt for 1 μg mL^−1^ of humic acids was observed.

### Analytical performance

In the presented work owing to using thin Millipore membrane as the carrier and the sample of diameter smaller than the spot size of X-ray beam a satisfactory signal-to-background ratio in XRF analysis was obtain. If the thickness of the carrier and the mass of the sample are too large the sample can not be considered thin, and consequently the matrix effects must be corrected. If the area of the sample is too large, only a small fraction of the extracted elements is excited, and as a result, a low intensity of fluorescent radiation is observed.

Analytical figures of merit of the method using ethylenediamine-modified graphene oxide as solid sorbent are presented in Table 1. The analytical curves for Fe(III), Co(II), Ni(II), Cu(II), Zn(II) and Pb(II) were linear up to 40 ng mL^−1^ with the correlation coefficients better than 0.99. The limits of detection were calculated from LOD = (3/k)(B/t)^1/2^, where k is the sensitivity of the method, B is the background count rate in counts s^−1^ and t is the counting time. As can be seen in Table 1, XRF measurement allows obtaining LODs in the range of 0.06–0.1 ng mL^−1^.

**Table 1:** The parameters characterizing the method

Analyte	Linearity range, ng mL^−1^	Equation (C in ng mL^−1^) (I in cps)	Correlation coefficient, R	LOD, ng mL^−1^	RMS, ng mL^−1^	RSD, %	Recovery, %
Fe	2–40	I = 1.341 × C + 5.3902	0.9991	0.07	0.646	4.1	90
Co	2–40	I = 1.413 × C + 4.4495	0.9997	0.10	0.370	4.8	91
Ni	2–40	I = 2.201 × C + 7.0342	0.9990	0.07	0.614	4.4	93
Cu	2–40	I = 2.460 × C + 5.0954	0.9978	0.08	0.814	4.5	96
Zn	2–40	I = 3.346 × C + 23.554	0.9985	0.06	1.045	5.0	95
Pb	2–40	I = 1.712 × C + 0.7857	0.9994	0.10	0.543	3.6	98

The average relative standard deviation (RSD) characterizing the precision of the method is ca. 4.4 %. The RMS (root mean square of the sum of the differences between the chemical values of the standard concentrations and the calculated values) characterizing the dispersion points around the straight line ranged from 0.37 to 1.045 ng mL^−1^.

### Analytical application

The accuracy of the method was verified by determining the elements in the standard reference materials LGC6016 (estuarine water) and BCR-610 (groundwater) as well as by recovery experiments. The results presented in Table 2 show very good agreement between certified and determined concentrations of nickel, copper and lead.

**Table 2:** Analysis of certified reference materials (*n* = 3)

CRM	Matrix	Analyte	Certified concentration, ng g^−1^	Determined concentration, ng g^−1^	Relative difference (%)
LGC6016	4700 μg g^−1^ Na,	Mn	976 ± 31	–	–
	570 μg g^−1^ Mg,	Cu	190 ± 4	184 ± 6	3.3
	220 μg g^−1^ Ca,	Ni	186 ± 3	179 ± 5	3.8
	180 μg g^−1^ K	Pb	196 ± 3	203 ± 5	3.4
BCR-610	Major and minor elements are not given in certificate	Al	159 ± 4	–	–
		Cu	45.7 ± 1.5	47.8 ± 1.3	4.5
		Pb	7.78 ± 0.13	7.4 ± 0.2	4.9
		Ni	22.6^a^	20.8 ± 0.8	8.1

^a^Approximate concentration based on the results from 3 laboratories

The developed method was used for the determination of Fe(III), Co(II), Ni(II), Cu(II), Zn(II) and Pb(II) in water samples from an industrial region of Poland. The results of EDXRF analysis as well as the recovery for the spiked samples, are given in Table 3. The determination of known added amounts of Fe(III), Co(II), Ni(II), Cu(II), Zn(II) and Pb(II) into water sample by EDXRF allows evaluating the accuracy of the method and the possible effects from real matrix. The concentrations of added to real water analytes were 10 ng mL^−1^ or 20 ng mL^−1^. The results presented in Table 3 show that the recoveries of determined elements are justifiable for trace analysis, in the range of 92–110 %.

**Table 3:** The results of EDXRF analysis of water and water samples spiked with known amounts of Fe(III), Co(II), Ni(II), Cu(II), Zn(II) and Pb(II)

Element	Spiked, ng mL^−1^	Determined, ng mL^−1^	Recovery, %
Fe	0	21.4 ± 0.8	–
	10	32.1 ± 0.9	107
	20	40.6 ± 1.8	96
Co	0	4.1 ± 0.2	–
	10	13.6 ± 0.3	95
	20	26.1 ± 1.1	110
Ni	0	< DL	–
	10	10.3 ± 0.5	103
	20	19 ± 0.7	96
Cu	0	10.1 ± 0.2	–
	20	20.8 ± 0.8	107
	20	31.2 ± 1.3	104
Zn	0	25.4 ± 0.9	–
	10	35.1 ± 1.3	97
	20	43.8 ± 1.7	92
Pb	0	7.7 ± 0.3	–
	10	18.5 ± 0.7	108
	20	28 ± 1.2	102

### Comparison of developed dispersive micro-solid phase extraction with other methods

Carbon-based nanomaterials have received much attention for their potential applications in preconcetration of trace elements. The summarized specific features of the developed method with comparison to others are given in the Table 4. The table includes the reported method and some recently published methods for the determination of Fe(III), Co(II), Ni(II), Cu(II), Zn(II) and Pb(II) using graphene (G) and graphene oxide (GO). The advantage of developed DMSPE in comparison with SPE is that a very small mass of nanomaterial, only 2 mg was used instead of 30–200 mg required in SPE [25, 26, 39, 40, 45, 46]. Because classical SPE relies on passing the liquid sample with analytes through a column containing an adsorbent very small particles of nanomaterial can cause high pressure in the SPE column and/or nanoparticles can escape from the SPE cartridge. For these reasons, using SPE column packed with highly hydrophilic sorbent like GO nanosheets is severely hampered or even impossible. Some authors, to overcome these difficulties, apply hollow fiber solid-phase microextraction [48] or covalently bind graphene oxide nanosheets to support [26]. This problem does not concern developed DMSPE method. Additionally GO-EDA unlike to graphene [42, 43] does not require the application of a chelating agent because of the presence of the nitrogen-containing groups on the surface. The developed method connected with EDXRF measurement is also characterized by excellent LOD at the level of ETAAS [41] and ICP-MS [48] techniques.

**Table 4:** Figures of merit of recently reported carbon-based nanomaterials for determination / preconcentration of Fe(III), Co(II), Ni(II), Cu(II), Zn(II) and Pb(II)

Sorbent	Mass [mg]	Method/Technique	Element	Analytical range, ng mL^−1^	LOD, ng mL^−1^	RSD, %	pH	Ref.
G/Co_3_O_4_	100	SPE/FAAS	Fe(III)	–	0.15	2.2	7.0	[39]
			Cu(II)		0.17	1.8		
			Pb(II)		0.81	1.9		
Amino/G	100	SPE/	Pb	–	1.1	1.2	7.0	[40]
Tri-amino/G		FAAS	Pb		0.9	1.7		
G/CL^a^	5	USA-DμSPE^b^/ETAAS	Pb	0.24–10.3	0.07	3.4	5.0	[41]
Dithizone/G	100	SPE/WDXRF	Co(II)	10–3000	1.30	14.1	10	[42]
			Ni(II)		1.10	15.6		
			Cd(II)		6.10	14.0		
			Pb(II)		2.00	8.3		
G/Triton X-100	0.8	DMSPE/EDXRF	Co(II)	5–100	0.08	2.7	5.0	[43]
			Ni(II)		0.07	2.6		
			Cu(II)		0.08	3.4		
			Pb(II)		0.20	2.8		
GO	0.5	DMSPE/EDXRF	Co(II)	5–100	0.50	4.25	5.0	[44]
			Ni(II)	5–100	0.70	4.52		
			Cu(II)	5–100	1.50	2.53		
			Zn(II)	10–100	1.80	5.07		
			Pb(II)	5–100	1.40	3.41		
GO/TiO_2_	–	on-line						
		SPE/ICP-OES	Cu(II)	1–1000	0.48	6.4	5.0	[45]
			Pb(II)	10–1000	2.64	9.8		
GO/MHE^c^	30	SPE/FAAS	Co(II)	3–215000	0.25	2.4	6.0	[46]
			Ni(II)	2–220000	0.18	2.1		
GO@SiO_2_	200	SPE/FAAS	Cu(II)	1.0–160	0.084	0.87	–	[26]
			Pb(II)	2.0–200	0.27	1.00		
GO-silica	–	HF/SPME^d^/ICP-MS	Co(II)	0.01–50	0.0004	7.0	5.0	[47]
			Ni(II)	0.1–50	0.020	5.6		
			Cu(II)	0.1–50	0.023	7.3		
			Pb(II)	0.1–50	0.028	4.6		
Fe_3_O_4_/GO	6.0	MSPE/ICP-MS	Co(II)	1.0–150	0.016	3.8	6.5	[48]
			Ni(II)		0.046	1.8		
			Cu(II)		0.395	3.5		
			Pb(II)		0.157	5.5		
MPPC^e^/GO	30	SPE/FAAS	Fe(III)	0.34–380	0.162	3.06	6.0	[25]
GO-EDA	2.0	DMSPE/EDXRF	Fe(III)	2.0–40	0.07	4.1	8.0	This work
			Co(II)		0.10	4.8		
			Ni(II)		0.07	4.4		
			Cu(II)		0.08	4.5		
			Zn(II)		0.06	5.0		
			Pb(II)		0.10	3.6		

^a^Clinoptilolite

^b^Ultrasound-assisted dispersivemicro solid phase extraction

^c^N-(5-methyl-2-hydroxyacetophenone)-N′-(2-hydroxyacetophenone) ethylene diamine

^d^Hollow fiber solid phase microextraction

^e^3-(1-Methyl-1H-pyrrol-2-yl)-1H-pyrazole-5-carboxylic acid

## Conclusions

A simple, fast and completely solvent free method using ethylenediamine-modified graphene oxide for the adsorption of Fe(III), Co(II), Ni(II), Cu(II), Zn(II) and Pb(II) has been developed. Ethylenediamine-modified graphene oxide as a sorbent can be readily obtained in most chemical laboratories, what greatly promotes its wide analytical application. Ethylenediamine-modified graphene oxide is particularly interesting for the preconcentration/removal of metal ions because of its extremely hydrophilic properties and the presence of functional groups containing nitrogen and oxygen atoms. These groups can efficiently bind the metal ions to form a metal complex through sharing an electron pair. Dispersive micro-solid phase extraction using ethylenediamine-modified graphene oxide as the preconcentration method together with EDXRF measurement allow simultaneous, multielemental and rapid analysis of the solid samples with good accuracy and precision, high recovery and low cost. Due to the elements sorbed on ethylenediamine-modified graphene oxide do not have to be eluated from the sorbent before the XRF measurement, the risk of loss of analytes and the contamination of the sample is reduced to the minimum. The samples can be stored and analyzed many times. The combination of the developed method and XRF spectrometry can be good alternative to other techniques, that are commonly used in the analysis of liquid samples but which are in need of the eluation of the analytes from the sorbent. The results indicate that the developed method can be hopeful for the determination of the trace elements not only in natural water but also in the samples containing the organic matter as well as in salinity solutions.

## Electronic supplementary material

ESM 1 (DOC 222821 kb)
