# Supplementary material for: Preconcentration of Fe(III), Co(II), Ni(II), Cu(II), Zn(II) and Pb(II) with ethylenediamine-modified graphene oxide
Source: Mikrochim Acta. 2015 Sep 7;183:231–40. doi: 10.1007/s00604-015-1629-y (PMC4701767; doi:10.1007/s00604-015-1629-y)
Supplement: Supplementary file 1 — (DOC 222821 kb) [file 604_2015_1629_MOESM1_ESM.doc]

# Supporting Information

**Preconcentration of Fe(III), Co(II), Ni(II), Cu(II), Zn(II) and Pb(II) with ethylenediamine-modified graphene oxide.**

**Beata Zawisza,*^a^ Anna Baranik^a^, Ewa Malicka^a^, Ewa Talik^b^ and Rafał Sitko^a^**

^a^ University of Silesia, Institute of Chemistry, ul. Szkolna 9, 40-006 Katowice, Poland

^b^ University of Silesia, Institute of Physics, ul. Uniwersytecka 4, 40-007 Katowice, Poland

* e-mail address: [beata.zawisza@us.edu.pl](mailto:beata.zawisza@us.edu.pl)

* telephone number: +48323592258

# Optimization of method

**The effect of the solution pH**

The effect of the solution pH on the preconcentration of metal ions on GO-EDA and on the extraction of analytes from water solutions was investigated within the pH range of 2 to 11.

Amine groups on the surface of GO-EDA are strongly reactive with metal ions. Nitrogen atoms hold free electron doublets that can react with metal cations. Due to the protonation of  the amine groups in acidic solutions, the worse results can be obtained in the acidic sample. Moreover, the adsorption depends also on the metal species present in the solution.  At pH 1−7, the predominant metal species are Fe(OH)^2+^, Co^2+^, Ni^2+^, Cu^2+^, Zn^2+^ and Pb^2+^. However, the protons in acid solution can protonate binding sites of the chelating molecules onto the surface of GO-EDA (the point of zero charge pH_PZC_ of carbon surface modified with EDA is ca. 5.91 [1]). In consequence, the positive charges generated on the GO-EDA surface (at pH <pH_PZC_) result in the electrostatic repulsion. In neutral and basic solution, the favorite mechanism is the reaction between the lone pair of electrons in nitrogen of EDA and metal ion, which results in formation of metal complex. However, the metal ions begin to form precipitates at pH 8−11 depending on the initial metal concentrations. As can be seen, in detail, in Fig. S1, the pH of the aqueous solution plays an important role to the adsorption of trace elements on GO-EDA in the presented study. With increasing pH, the adsorption of determined elements on GO-EDA increases at pH ranging from 2 to 7. Then, the adsorption percentage accomplishes the maximum value and remains constant at pH 7 - 10, and at last at pH>10 decreases, because of formation of soluble anionic forms of determined elements. Higher than 90% of Fe(III), Co(II), Ni(II), Cu(II), Zn(II) and Pb(II) is adsorbed on GO-EDA at pH >7. When pH value is higher than 7 the negative charged surface of GO-EDA can also provide electrostatic attraction that are favorable for the adsorption of cationic species, thus the adsorption of determined ions on GO-EDA increases abruptly. Thus, as mentioned above, in the solution of pH=8, in metal-GO-EDA combination, amine groups are mainly responsible for the uptake of metal cations by a chelation mechanism. But, it must be also emphasized, that the preconcentration of trace elements is possibly accomplished by simultaneous precipitation and adsorption of elements on GO-EDA.

Fig. S1. The influence of pH on the recovery of Fe(III), Co(II), Ni(II), Cu(II), Zn(II) and Pb(II) ions on ethylenediamine-modified graphene oxide; conditions: C = 0.02 mg L^−1^, C_GO-EDA_ = 0.04 g L^−1^, stirring time = 5 min, the error bars correspond to one standard deviation, n = 3.

The presented results show that the preconcentration process for Fe, Co, Ni, Cu, Zn and Pb ions from the solution of pH=8 is achieved with high recoveries. These elements can be determined with recoveries of 90±9%, 91±6%, 93±8%, 96±10%, 95±7%, and 98±8% for Fe, Co, Ni, Cu, Zn and Pb, respectively. In consequence of carried out study pH=8 was chosen as the optimum pH for further studies.

**The effect of the concentration of ethylenediamine-modified graphene oxide**

The mass of the sorbent is highly connected with the sample volume. The effect of the concentration of ethylenediamine-modified graphene oxide on the recoveries of determined elements was investigated using varying concentration of ethylenediamine-modified graphene oxide from 0.01 to 0.4 g L^-1^. Each solution contained 1 μg of Fe(III), Co(II), Ni(II), Cu(II), Zn(II) and Pb(II) and 2 mg or 4 mg of GO-EDA. The results, which are illustrated in Fig. S2 show that the recoveries of Fe(III) and Cu(II) remained constant within 0.01-0.4 g L^-1^ of the concentration of ethylenediamine-modified graphene oxide, the recoveries of Co(II) and Pb(II), remained constant within the concentration of 0.04-0.2 g L^-1^, and the recoveries of Ni(II) and Zn(II) remained constant within 0.04-0.4 g L^-1^ of ethylenediamine-modified graphene oxide concentration. Finally, the concentration of 0.04 g L^-1^ of ethylenediamine-modified graphene oxide (sample volume of 50 mL and 2 mg of GO-EDA) was chosen for the subsequent experiments.

Fig. S2. The influence of concentration of ethylenediamine-modified graphene oxide on the recovery of Fe(III), Co(II), Ni(II), Cu(II), Zn(II) and Pb(II) ions on GO-EDA; conditions: C_GO-EDA_ = 0.04 g L^−1^ or 0.08 g L^−1^, pH=8, stirring time = 5 min, the error bars correspond to one standard deviation, n = 3.

**The effect of stirring time**

Fig. S3. The influence of the sorption time on the recovery of Fe(III), Co(II), Ni(II), Cu(II), Zn(II) and Pb(II) ions on ethylenediamine-modified graphene oxide; conditions: C_GO-EDA_= 0.02 mg L^−1^, C_GO-EDA_ = 0.04 g L^−1^, pH=8, the error bars correspond to one standard deviation, n = 3.

The time of stirring is the measure of the contact time between the GO-EDA and the sample solution. This effect was investigated using varying time from 5 to 120 min, 2 mg of GO-EDA and 50 mL sample containing 1 μg of Fe(III), Co(II), Ni(II), Cu(II), Zn(II) and Pb(II). As shown in Fig. S3, time variation in the range of 5–120 min does not influence the high recoveries of all determined elements onto GO-EDA. The high recoveries obtained even after 5 min of stirring indicate the instant reaction between the ions of determined elements and the functional groups of GO-EDA. Such instant reaction between the ions and GO-EDA resulted from the fact that the used sorbent was dispersed in the whole volume of aqueous samples. Finally, the analyzed solutions were stirred for 5 min in the subsequent experiments.

**The effect of matrix elements**

The effects of common coexisting ions in different types of water, such as Na(I), K(I), Mg(II), Ca(II), on the preconcentration of Fe(III), Co(II), Ni(II), Cu(II), Zn(II) and Pb(II) on GO-EDA were investigated. The results of this investigation are presented in the Figure S4.

|  |  |
| --- | --- |
|  |  |
|  |  |

Fig. S4. The influence of interfering ions on the recovery of Fe(III), Co(II), Ni(II), Cu(II), Zn(II) and Pb(II) ions on ethylenediamine-modified graphene oxide; conditions: C = 0.02 mg L^−1^, C_GO-EDA_ = 0.04 g L^−1^, pH=8, stirring time = 5 min, the error bars correspond to one standard deviation, n = 3.

In some types of water the concentration of Fe(III) can be also high. Thus, the influence of the high concentration of Fe(III) on the recovery of Co(II), Ni(II), Cu(II), Zn(II) and Pb(II) on GO-EDA was investigated in detail. The obtained results are showed in Fig. S5.

Fig. S5. The influence of Fe(III) on the recovery of Co(II), Ni(II), Cu(II), Zn(II) and Pb(II) ions on GO-EDA; conditions: C = 0.02 mg L^−1^, C_GO-EDA_ = 0.04 g L^−1^, pH=8, stirring time = 5 min, the error bars correspond to one standard deviation, n = 3.

The influence of HA on the preconcentration of heavy metals by GO-EDA was investigated. The results of this investigation are presented in the Fig. S6.

Fig. S6. The influence of humic acid on the recovery of Fe(III), Co(II), Ni(II), Cu(II), Zn(II) and Pb(II) ions on GO-EDA; conditions: C = 0.02 mg L^−1^, C_GO-EDA_ = 0.04 g L^−1^, pH=8, stirring time = 5 min, the error bars correspond to one standard deviation, n = 3.

**References**

1. Vukovic G, Marinkovic A, Colib M, Ristic M, Aleksic R, Peric-Grujic A, Uskokovic P (2010) Removal of cadmium from aqueous solutions by oxidized and ethylenediamine-functionalized multi-walled carbon nanotubes. Chem Eng J 157:238–248
